# Mortality risk and causes of death in patients with non-cystic fibrosis bronchiectasis

**DOI:** 10.1186/s12931-019-1243-3

**Published:** 2019-12-03

**Authors:** Sooim Sin, Seo Young Yun, Jee Min Kim, Chang Min Park, Jaeyoung Cho, Sun Mi Choi, Jinwoo Lee, Young Sik Park, Sang-Min Lee, Chul-Gyu Yoo, Young Whan Kim, Sung Koo Han, Chang-Hoon Lee

**Affiliations:** 1 Division of Pulmonary and Critical Care Medicine, Department of Internal Medicine, Seoul National University Hospital, 101 Daehak-Ro Jongno-Gu, Seoul, 03080 Republic of Korea; 2 Kangwon National University College of Medicine, Chuncheon, Republic of Korea; 3 Division of Pulmonary and Critical Care Medicine, Seoul Metropolitan Government-Seoul National University Boramae Medical Centre, Seoul, Republic of Korea; 4 Division of Pulmonary and Critical Care Medicine, Department of Internal Medicine, National Medical Centre, Seoul, Republic of Korea; 5 Department of Radiology, Seoul National University College of Medicine, Seoul, Republic of Korea; 6 Department of Internal Medicine, Seoul National University College of Medicine, Seoul, Republic of Korea

**Keywords:** Bronchiectasis, Mortality, Cause of death

## Abstract

**Background:**

All-cause mortality risk and causes of death in bronchiectasis patients have not been fully investigated. The aim of this study was to compare the mortality risk and causes of death between individuals with bronchiectasis and those without bronchiectasis.

**Methods:**

Patients with or without bronchiectasis determined based on chest computed tomography (CT) at one centre between 2005 and 2016 were enrolled. Among the patients without bronchiectasis, a control group was selected after applying additional exclusion criteria. We compared the mortality risk and causes of death between the bronchiectasis and control groups without lung disease. Subgroup analyses were also performed according to identification of *Pseudomonas* or non-tuberculous mycobacteria, airflow limitation, and smoking status.

**Results:**

Of the total 217,702 patients who underwent chest CT, 18,134 bronchiectasis patients and 90,313 non-bronchiectasis patients were included. The all-cause mortality rate in the bronchiectasis group was 1608.8 per 100,000 person-years (95% confidence interval (CI), 1531.5–1690.0), which was higher than that in the control group (133.5 per 100,000 person-years; 95% CI, 124.1–143.8; *P* < 0.001). The bronchiectasis group had higher all-cause (adjusted hazard ratio (aHR), 1.26; 95% CI, 1.09–1.47), respiratory (aHR, 3.49; 95% CI, 2.21–5.51), and lung cancer-related (aHR, 3.48; 95% CI, 2.33–5.22) mortality risks than the control group. In subgroup analysis, patients with airflow limitation and ever smokers showed higher all-cause mortality risk among bronchiectasis patients. Therefore, we observed significant interrelation between bronchiectasis and smoking, concerning the risks of all-cause mortality (*P* for multiplicative interaction, 0.030, RERI, 0.432; 95% CI, 0.097–0.769) and lung cancer-related mortality (RERI, 8.68; 95% CI, 1.631–15.736).

**Conclusion:**

Individuals with bronchiectasis had a higher risk of all-cause, respiratory, and lung cancer-related mortality compared to control group. The risk of all-cause mortality was more prominent in those with airflow limitation and in ever smokers.

## Background

Non-cystic fibrosis bronchiectasis (hereafter referred to as bronchiectasis) is characterised by the irreversible dilation of the bronchial lumen caused by recurrent or chronic infection and inflammation, which is not related to cystic fibrosis, and clinically it causes chronic cough, sputum, and respiratory infection [1]. Not only is bronchiectasis a chronic respiratory disease with substantial disease burden worldwide, but it is also associated with significant morbidity and hospitalization [2, 3]. According to the data from health-care claims in the United States, the prevalence of bronchiectasis was over 500 per 100,000 persons in 2000 and over 800 per 100,000 persons in 2007 which showed a continuous increase over time [3, 4]. Moreover, the increased demand for medical resources for patients with bronchiectasis cannot be ignored since annually, patients with bronchiectasis demonstrated significantly increased medical costs, outpatient visits, and a higher admission rate than normal populations [5, 6]. However, bronchiectasis has been an underestimated disease in spite of its rampancy and the increasing economic burden of the disease as well as its considerable morbidity and mortality [3–10]. Although there have been several studies reporting mortality in patients with bronchiectasis, several issues remain unaddressed. Previous studies have reported on the death rates of patients with bronchiectasis, but those had a wide range (10–42% at 4–5 years and 22–30% at 34–35 years) [9, 11–14]. Furthermore, it has not been verified if bronchiectasis leads to a higher risk for early death. Previous studies have not included control groups to compare the mortality risk to those with bronchiectasis. Moreover, until now there has been a lack of studies regarding cause of death in patients with bronchiectasis, and no large-scale study has been conducted in Asia, where bronchiectasis is more common than in western countries.

The main objective of the present study was to investigate all-cause mortality risk and causes of death in patients with bronchiectasis compared with those in the non-bronchiectatic control group without lung disease. We also attempted to identify higher mortality risk subgroups among those with bronchiectasis.

## Methods

### Study design and participants

This retrospective cohort study included participants aged ≥20 years who underwent chest computed tomography (CT) at the Seoul National University Hospital between January 1, 2005 and December 31, 2016. The diagnosis of bronchiectasis was determined based on the CT report, which was assessed by radiologists. Index date was the time when these patients underwent chest CT. We excluded those regarded to have previously undergone lung resection surgery (such as pneumonectomy, lobectomy, segmentectomy, wedge resection, metastasectomy, or video-assisted thoracoscopic surgery [VATS] lung biopsy) on the chest CT or those with significant non-respiratory (malignancy, liver cirrhosis, chronic kidney disease, heart failure, acquired immune deficiency syndrome, connective tissue disease, organ transplantation) or respiratory diseases (interstitial lung disease, pneumoconiosis, pleurisy, empyema, sarcoidosis, kyphosis, or tuberculous-destroyed lung) which may affect the prognosis. The identification of comorbidities or exclusion criteria was based on ICD-10 codes registered within two years before and after the index date. Those with traction bronchiectasis were also excluded. To ensure appropriate selection of individuals in the non-bronchiectasis control group, we applied the following additional exclusion criteria: 1) isolation of nontuberculous mycobacteria (NTM) from a respiratory specimen at least once; 2) a forced expiratory volume in one second (FEV_1_)/forced vital capacity (FVC) ratio < 0.7 or FEV_1_ < 80% predicted value; or 3) other abnormalities on CT such as pneumonia, active pulmonary tuberculosis, pneumothorax, pleural effusion, pericardial effusion, pulmonary embolism, embolization in coronary artery, aorta or bronchial artery, pleural calcification, or haemoptysis. In the bronchiectasis group, patients were divided into two categories according to the isolation of NTM, the isolation of *Pseudomonas*, smoking status, and airflow limitation defined as a FEV_1_/FVC of less than 0.7. During the study period, patients with any evidence of NTM or *Pseudomonas* were classified into NTM or *Pseudomonas* subgroups. This study was approved by the Institutional Review Board of the Seoul National University Hospital (IRB No. H-1607-037-774) and conformed to the tenets of the Declaration of Helsinki. Informed consent was waived due to its retrospective nature.

### Statistical analysis

Baseline demographic data, comorbidities, smoking history, sputum culture results, and lung function results were collected by using data based on electronic medical records (EMRs). Student’s *t*-test or analysis of variance was used for between-group comparisons of demographics involving continuous variables, and the chi-square test for those involving categorical variables. Mortality and cause of death before 2016 December 31 was determined based on the mortality data from the Korean National Statistical Office. Cox proportional hazard regression analysis was used to present adjusted hazard ratios (aHR) and their 95% confidence intervals (CI). In addition, the proportion of mortality risks attributable to risk factors (population attributable risk fraction, PAF) and their 95% CIs were estimated. *P*-values for multiplicative interactions, as well as relative excess risk due to interaction (RERI) and attributable proportion (AP) for additive interactions were evaluated, along with their 95% CIs. *P*-values less than 0.05 were considered statistically significant. Data analyses were conducted using Stata14.2 (Stata Corp, College station, Texas).

## Results

### Baseline characteristics

**Fig. 1:**
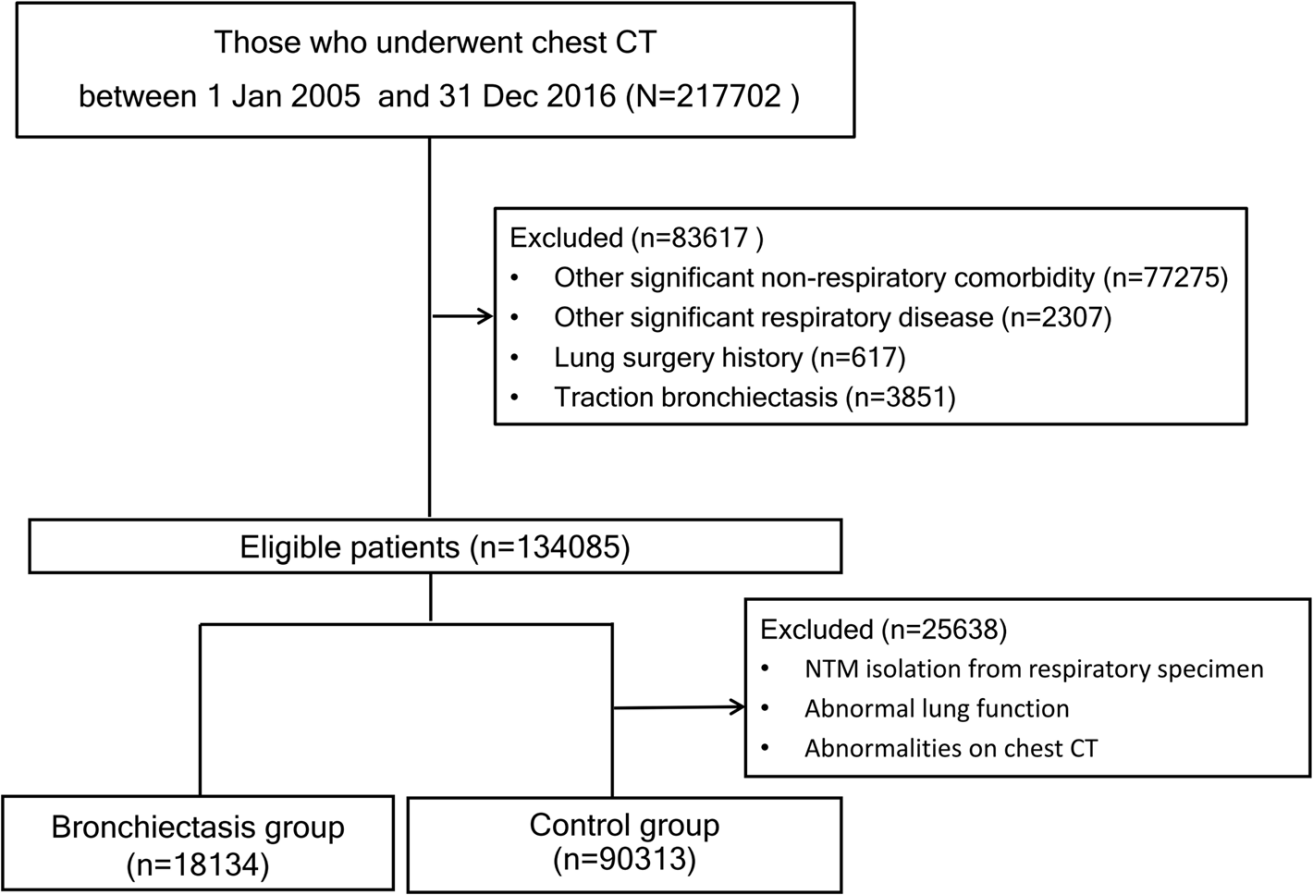
Flowchart for the study population

We initially identified 217,702 individuals who underwent a chest CT scan during the study period. Among those, 18,134 participants with bronchiectasis and 90,313 non-bronchiectatic control participants were included (Fig. 1). The baseline demographics of the subjects are shown in Table 1. The bronchiectasis group was older, had more females, and had a high proportion of comorbidities and poorer lung function.

**Table 1:** Baseline characteristics of participants

Variables	Control group (*n* = 90,313)	Bronchiectasis group (*n* = 18,134)	*P*-value
Age, years	51.5 ± 11.9	59.6 ± 11.7	< 0.001
Male	52,631 (58.3)	8654 (47.7)	< 0.001
BMI, kg/m^2^	23.8 ± 3.1	22.7 ± 3.3	< 0.001
Smoking			< 0.001
Never	15,931 (17.6)	5020 (27.7)	
Ever	10,264 (11.4)	2859 (15.8)	
Comorbidities			
Hypertension	9496 (10.5)	2194 (12.1)	< 0.001
Diabetes	6249 (6.9)	1552 (8.6)	< 0.001
Dyslipidaemia	13,707 (15.2)	2269 (12.5)	< 0.001
COPD	1882 (2.1)	16,498 (9.0)	< 0.001
Asthma	2372 (2.6)	1445 (8.0)	< 0.001
Baseline PFT, n	60,854	10,279	
FEV_1_ (L)	3.1 ± 0.7	2.3 ± 0.8	< 0.001
FEV_1_ (% predicted)	98 ± 11	92 ± 16	< 0.001
FVC (L)	3.8 ± 0.9	3.1 ± 0.9	< 0.001
FVC (% predicted)	98 ± 11	92 ± 16	< 0.001
FEV_1_: FVC ratio (%)	81 ± 5	74 ± 12	< 0.001

Categorical variables are expressed as number (%); continuous variables are expressed as mean +/− standard deviation.

*BMI* body mass index, *COPD* chronic obstructive pulmonary disease, *FVC* functional vital capacity, *FEV1* forced expiratory volume in 1 s, *PFT* pulmonary function test

### Mortality risk

**Fig. 2:**
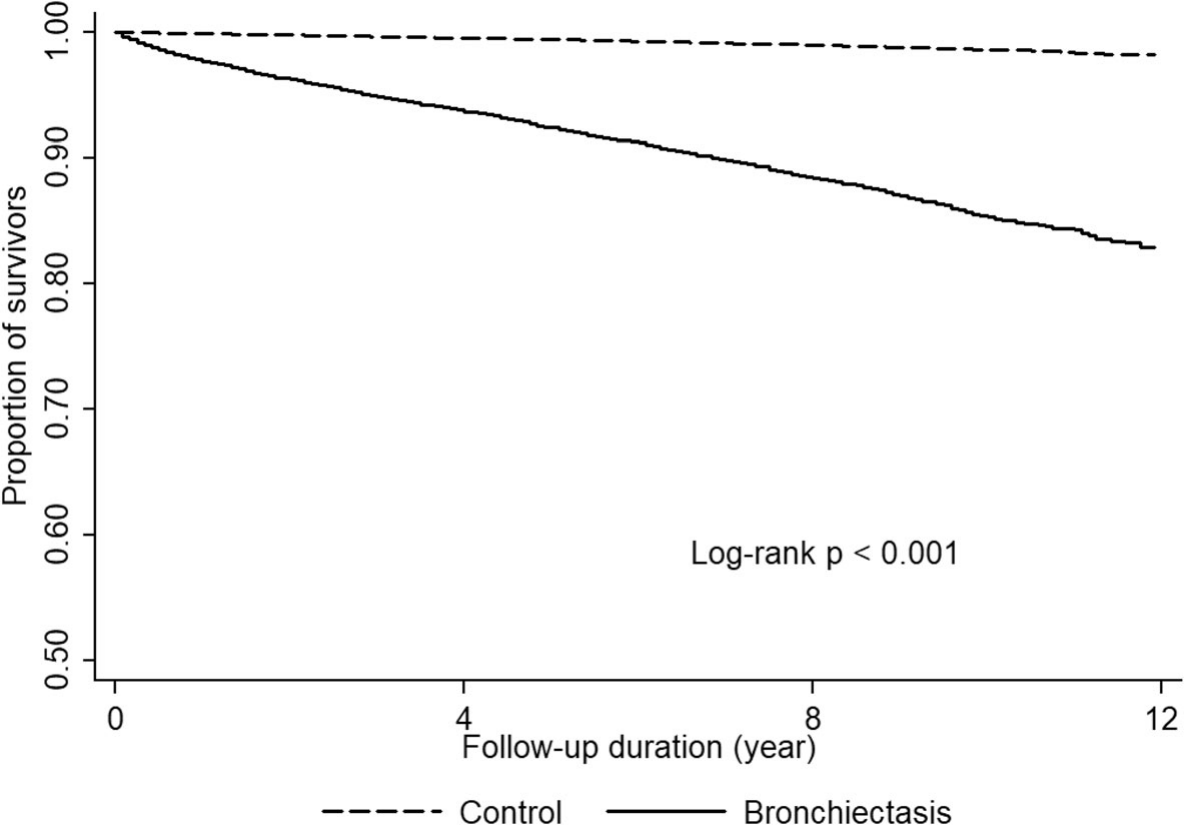
Bronchiectasis increased risk of all-cause mortality in comparison with the control group

In total, 1653 (9.12%) deaths in the bronchiectasis group and 714 (0.79%) deaths in the control group were observed during 5.9 ± 3.4 and 5.4 ± 3.5 years of mean follow up period, respectively. The all-cause mortality rate in the bronchiectasis group was 1608.8 per 100,000 person-years (95% CI, 1531.5–1690.0) which was higher than that of the control group (133.5 per 100,000 person-years; 95% CI, 124.1–143.8) (*P* < 0.001) (Fig. 2). Multivariable cox proportional hazard regression showed that bronchiectasis remained independently associated with a higher all-cause mortality risk (aHR, 1.22; 95% CI, 1.02–1.46) compared with the control group even after adjustment for age, sex, BMI, smoking history, presence of diabetes mellitus, hypertension, and dyslipidaemia, and baseline FEV_1_(pred. %) (Table 2). In addition, bronchiectasis and smoking per se were accountable for 9.3% (population attributable fraction (PAF), 0.093; 95% CI, 0.012–0.167) and 9.2% (PAF, 0.092; 95% CI, 0.029–0.151) of all-cause mortality, respectively. Among participants with bronchiectasis, smoking was associated with 14.0% (PAF, 0.140; 95% CI 0.042–0.228) of all-cause mortality, while in the control group, the percentage attributed to smoking was 5.7% (PAF, 0.057; 95% CI, − 0.025-0.0133). We found multiplicative (*P* for interaction, 0.030) and additive interaction (RERI, 0.432; 95% CI, 0.097–0.769; AP, 0.274, 95% CI, 0.080–0.469) between the two factors, regarding the risk of all-cause mortality. Patients with both bronchiectasis and smoking history had the highest risk of all-cause mortality among four subgroups, categorized according to bronchiectasis and smoking history (aHRs on all-cause mortality (95% CI); 1.07 (0.86–1.34), 1.07 (0.87–1.32), 1.58 (1.25–1.99) for ever-smokers in the control group, never-smoker bronchiectasis patients and ever-smoker bronchiectasis patients, respectively) (Additional file 3).

**Table 2:** Bronchiectasis is an independent risk factor of increased all-cause mortality risk. (multivariable cox proportional hazard regression analysis)

	aHR^a^	95% CI	*P*-value
Age, year	1.09	1.08–1.10	< 0.001
BMI, kg/m^2^	0.90	0.88–0.92	< 0.001
Female	0.79	0.67–0.94	0.008
Ever smoker	1.27	1.08–1.49	0.004
Hypertension	0.85	0.70–1.02	0.085
Diabetes	1.16	0.95–1.40	0.145
Dyslipidaemia	0.35	0.27–0.45	< 0.001
FEV1, % pred.	0.98	0.98–0.99	< 0.001
Bronchiectasis	1.22	1.02–1.46	0.028

^a^Adjusted by Age, BMI, sex, smoking status, hypertension, diabetes, dyslipidaemia, FEV1 (% pred.), bronchiectasis

*CI* confidence interval, *BMI* body mass index, *COPD* chronic obstructive pulmonary disease, *FVC* functional vital capacity, *FEV1* forced expiratory volume in 1 s

### Causes of death

**Fig. 3:**
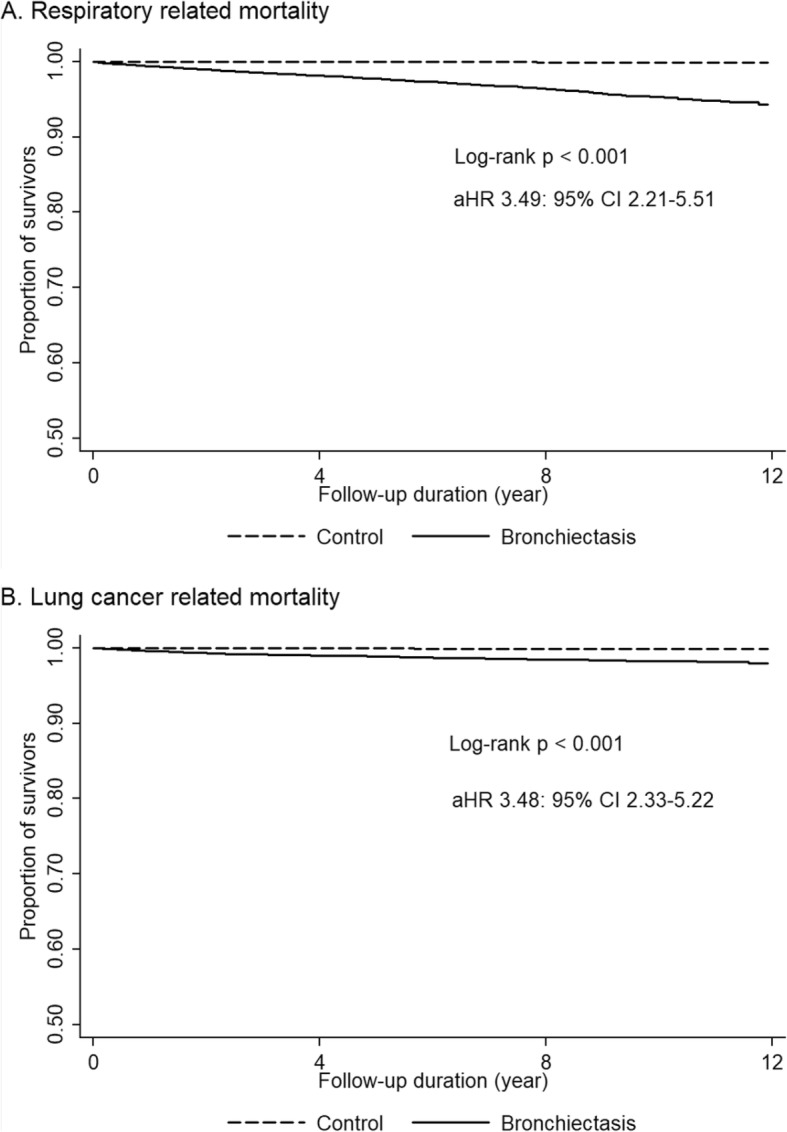
Bronchiectasis increased risk of respiratory related death and lung cancer-related death compared with the control group. **a** Comparision of respiratory related mortality between bronchiectasis patients and control population **b** Comparison of lung cancer related mortality between bronchiectasis patients and control population

The major causes of death in the bronchiectasis group were malignancy (31.2%), respiratory related (30.6%), neurological (9.0%), and cardiovascular death (7.1%). In the control group, malignancy (52.8%), cardiovascular (9.7%), neurological (9.2%), and suicide (9.1%) were major causes of death, in that order. Respiratory related deaths made up a small percentage (4.9%) of the total deaths in the control group (Table 3). Cox regression analysis showed bronchiectasis was significantly associated with increased respiratory death (aHR, 3.49; 95%; CI, 2.21–5.51) and lung cancer-related death (aHR, 3.36; 95% CI, 2.18–5.18) (Fig. 3). An additive interaction (RERI, 8.68, 95% CI, 1.631–15.736; AP, 0.586, 95% CI, 0.398–0.775) was observed between bronchiectasis and smoking, concerning the risk of lung cancer-related mortality. Bronchiectasis was not significantly related to other causes except for other cancer-related deaths compared with the control group.

**Table 3:** Causes of death in the control group versus the bronchiectasis group

Cause of death	Control group (total = 90,313)	Bronchiectasis group (total = 18,134)
Respiratory	35 (4.9)	505 (30.6)
All cancer	377 (52.8)	515 (31.2)
Lung cancer	52 (7.3)	205 (12.4)
Other cancer	325 (45.5)	310 (18.8)
Neurologic	66 (9.2)	148 (9.0)
Cardiologic	69 (9.7)	118 (7.1)
Trauma	40 (5.6)	46 (2.8)
Suicide	65 (9.1)	39 (2.4)
Others	62 (8.7)	282 (17.1)
Total	714	1653

### Risk factor of all-cause mortality in bronchiectasis

**Fig. 4:**
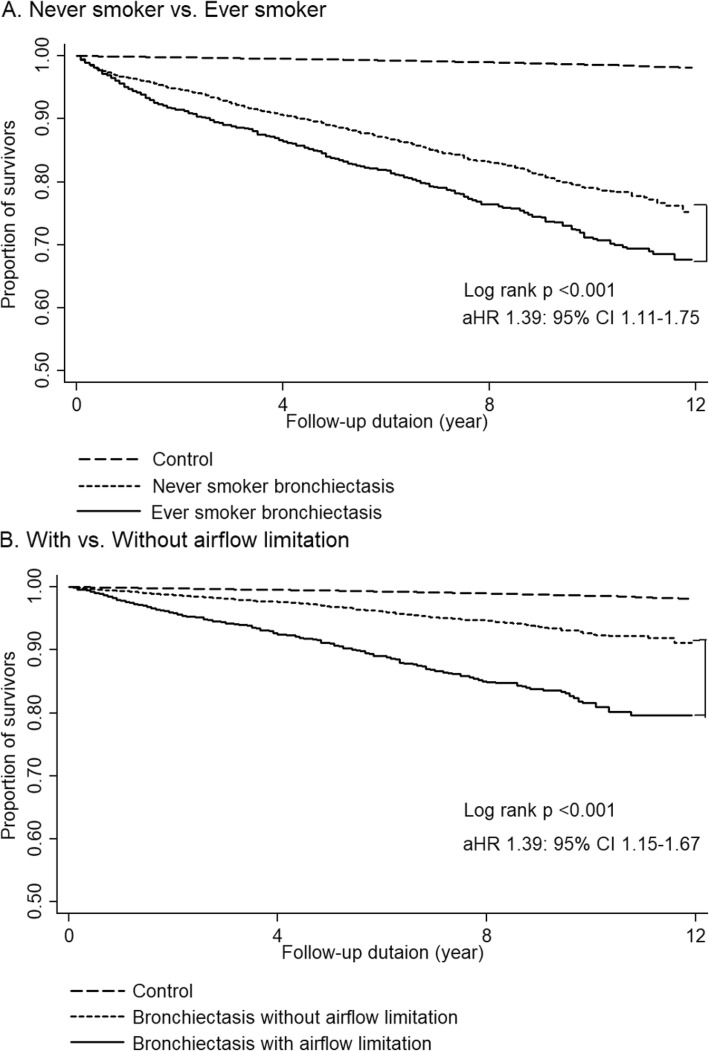
All-cause mortality risk according to additional risk factors. **a** Comparison of all-cause mortality risk between never smoker bronchiectasis patients versus ever smoker bronchiectasis patients **b** Comparision of all-cause mortality risk between bronchiectasis patients with airflow limitation versus bronchiectasis patients without airflow limitation

Among 6957 bronchiectasis patients whose respiratory specimen was tested for acid-fast bacteria (AFB) at least once during the study period, NTM was isolated in 1740 (25%). Additionally, among 4000 bronchiectasis patients whose respiratory specimen was tested for bacteria at least once during the study period, *Pseudomonas* was isolated in 378 (9.5%). Among patients with available lung function data, 2493 (24.3%) exhibited airflow limitations and among patients with available smoking history, 2859 (36.3%) were ever-smokers. According to multivariate Cox regression analysis, which was performed to evaluate the factors related to increased mortality in patients with bronchiectasis, airflow limitation and a history of smoking were significantly associated with increased mortality (aHR, 1.39; 95% CI, 1.15–1.67; aHR, 1.39; 95% CI, 1.11–1.75) (Fig. 4, Additional file 1 and Additional file 2).

## Discussion

The principal finding of the current study was that patients with bronchiectasis showed an increased risk of all-cause death, respiratory related death, and lung cancer-related death compared to control population without lung disease. Furthermore, all-cause mortality was higher in those with airflow limitation and in ever smokers among bronchiectasis patients. We also observed significant interactions between bronchiectasis and smoking regarding the risk of mortality.

The strengths of the current study are as follows. This was the first study to evaluate the association between bronchiectasis with increased mortality related to respiratory and lung cancer compared with a control population. In addition, to our knowledge, the present study has analysed the largest population ever, including 108,447 persons. Furthermore, there has not been a study about mortality and causes of death in Asian bronchiectasis patients completed previously.

Prior to our study, several studies investigated the mortality of bronchiectasis patients. However, there is a lack of consistency in the mortality of patients with bronchiectasis [9, 11–14]. The current study included a large number of bronchiectasis patients, with 9.12% mortality during the 5.8 ± 3.4 years of mean follow up period. This result is in line with previous studies, though slightly lower. We selected bronchiectasis patients by chest CT which was performed for various purposes, contrary to other studies which had enrolled patients according to the ICD code or the diagnosis from the respiratory clinic [9, 10, 12, 15, 16]. Therefore, our inclusion criteria may have enabled us to further characterise the natural course of bronchiectasis patients, even for those with mild symptoms, who rarely visit the respiratory clinics of tertiary hospitals. Furthermore, patients with milder disease, due to the strict inclusion criteria not replicated in other studies, may have influenced the results. In fact, the baseline lung function of patients in the current study was higher than those in previous studies; additionally, other studies did not use exclusion criteria with regard to comorbidities [9–12]. Another possible explanation is that there has been a lack of studies analysing Asian bronchiectasis patients, and hence, this result may represent the characteristics of the Asian population. Nevertheless, further research is needed.

The present study showed that the all-cause mortality risk of the bronchiectasis group was significantly higher than that of the control group. In total, 1475.3 more deaths per 100,000 person-years were observed than in the control group. Additionally, we showed the substantial attribution of mortality to bronchiectasis (PAF, 9.3%), which is comparable to the percentage attributed to smoking (PAF, 9.2%). In our study, respiratory related death could be one major cause that is responsible for this excess mortality risk. Cole’s vicious cycle model [17] is the most generally accepted concept in the pathogenesis of bronchiectasis. According to Cole’s vicious cycle, structural abnormalities, chronic inflammation, and recurrent infections continuously cause respiratory infections, acute exacerbation, and airflow limitation in patients throughout their lifetime, which induces premature death associated with respiratory causes [1, 18]. In fact, respiratory related deaths have been known to be a major cause of death in bronchiectasis [9, 19, 20]. In addition, to our knowledge, we reported here for the first time the higher risk of lung cancer-related mortality in those with bronchiectasis even after adjustment of smoking history, although there are several studies that have investigated the association of bronchiectasis with the incidence of lung cancer in Asian populations [21, 22]. Chronic inflammation and recurrent infections, which are generally accepted mechanisms for cancer development [23, 24], may contribute to the development of lung cancer in bronchiectasis patients; however, the exact biological mechanism needs further clarification. Interestingly, we found a statistically significant interaction between bronchiectasis and smoking, the well-known risk factor for lung cancer [25], concerning the risk of lung cancer-related mortality. Ever-smoker bronchiectasis patients exhibited the highest risk for lung cancer-related mortality. This interaction has not been reported before; however, further studies are needed to validate this finding, and reports related to the underlying mechanism are expected.

In previous studies, some factors associated with increased mortality or poor prognoses were identified in patients with bronchiectasis. Older age [9, 12, 13, 26], male [12, 20], smoking history [9], low BMI [13], isolation of *Pseudomonas* [12, 27, 28], and lung function measurements representing airflow limitation [12, 14] are some factors. Among those, we evaluated the association of mortality with factors such as airflow limitation (FEV_1_/FVC < 0.7), smoking history, and the isolation of NTM or *Pseudomonas* in a respiratory specimen. All four factors were significantly associated with increased mortality in patients with bronchiectasis. However, only airflow limitation and smoking history remained as significant factors and were associated with increased mortality in the multivariate analysis. Contrary to the considerable amount of data available from cystic fibrosis bronchiectasis patients, data from patients with non-cystic fibrosis bronchiectasis related to colonization by *Pseudomonas* is limited. It has been known that colonization by *Pseudomonas* is associated with an increased risk of mortality in cystic fibrosis bronchiectasis patients [29–31]. However, there are contradictory data in non-cystic fibrosis bronchiectasis patients [9, 10, 12, 15, 28]. Additionally, although the isolation of NTM in a respiratory specimen is not unusual [32, 33], its impact on mortality of NTM isolation in non-cystic fibrosis bronchiectasis patients remains unknown. Here, the results of the multivariable cox regression analysis adjusted for age, sex, BMI, and baseline FEV_1_ suggest that colonization by *Pseudomonas* (aHR, 1.04; 95% CI, 0.74–1.45) or NTM (aHR, 0.83; 95% CI, 0.64–1.08) may not be an independent risk factor related to the increased risk of mortality in non-cystic fibrosis bronchiectasis patients. However, in our study, sputum collection for *Pseudomonas* and NTM culture was conducted in only 22.1 and 38.4% of the participants, respectively. This should be addressed since it leads to a limited interpretation of the results.

We acknowledge several limitations in this study. Firstly, the control group in the current study was from hospital patients and not community controls. Hospital controls are more likely to have health related problems [34, 35] which could attenuate the difference in mortality risk between the control and patient groups. However, because community controls rarely undergo chest CT, which is the gold standard of diagnosis for bronchiectasis [36], we selected hospital controls. To address this limitation, we excluded patients with not only significant respiratory and non-respiratory comorbidities, but also used additional exclusion criteria. Furthermore, we adjusted various covariates including age, BMI, sex, smoking history, comorbidities, and baseline lung function to minimize bias.

## Conclusion

In conclusion, the current study showed that bronchiectasis is significantly associated with an increased risk of all-cause mortality, respiratory death, and lung cancer-related death compared with control population without lung disease. Physicians should pay more attention to those with airflow limitations and to smokers among bronchiectasis patients.

## Supplementary information

**Additional file 1.** Survival curves for all-cause mortality according to risk factors in the bronchiectasis group

**Additional file 2.** Adjusted HR and 95% CI of each risk factor from multivariate Cox regression analysis

**Additional file 3.** Survival curves for the combination of bronchiectasis and smoking (TIFF). aHRs on all-cause mortality (95% CI) were 1.07 (0.86–1.34), 1.07 (0.87–1.32), 1.58 (1.25–1.99), for ever-smokers in the control group, never-smoker bronchiectasis patients and ever-smoker bronchiectasis patients, respectively (*P* for multiplicative interaction = 0.030; RERI, 0.432; 95% CI, 0.097–0.769; AP, 0.274, 95% CI, 0.080–0.469). aHRs on lung cancer-related mortality (95% CI) were 4.09 (2.09–8.12), 3.01 (1.57–5.76), 14.8 (7.66–28.61), for ever-smokers in the control group, never-smoker bronchiectasis patients and ever-smoker bronchiectasis patients, respectively (RERI, 8.68, 95% CI, 1.631–15.736; AP, 0.586, 95% CI, 0.398–0.775)

## Data Availability

The datasets used and/or analysed during the current study are available from the corresponding author on reasonable request.

## Abbreviations

aHR: Adjusted hazard ratio
AP: Attributable proportion
BMI: Body mass index
CI: Confidence interval
COPD: Chronic obstructive pulmonary disease
CT: Computed tomography
EMR: Electronic medical records
FEV_1_: Forced expiratory volume in 1 s
FVC: Functional vital capacity
NTM: Nontuberculous mycobacteria
PAF: Population attributable risk fraction
PFT: Pulmonary function test
RERI: Relative excess risk due to interaction.
