# Supplementary material for: Mortality risk and causes of death in patients with non-cystic fibrosis bronchiectasis
Source: Respir Res. 2019 Dec 3;20:271. doi: 10.1186/s12931-019-1243-3 (PMC6889428; doi:10.1186/s12931-019-1243-3)
Supplement: Supplementary file 2 — Additional file 2. Adjusted HR and 95% CI of each risk factor from multivariate Cox regression analysis [file 12931_2019_1243_MOESM2_ESM.docx]

**Additional file 3.** Adjusted HR and 95% CI of each risk factor from multivariate Cox regression analysis

|  | **aHR** | **95% CI** | ***P*-value** |
| --- | --- | --- | --- |
| Ever-smoker | 1.39 | 1.11-1.75 | 0.004 |
| Airflow limitation | 1.39 | 1.15-1.67 | 0.001 |
| *Pseudomonas* isolation | 1.04 | 0.74-1.45 | 0.822 |
| NTM isolation | 0.83 | 0.64-1.08 | 0.161 |

aHR: adjusted hazard ratio; CI: confidence interval; HR: hazard ratio
